# Salivary vascular growth factor responses to prolonged and interrupted sitting in young, healthy adults

**DOI:** 10.14814/phy2.70798

**Published:** 2026-02-23

**Authors:** Alicia Kollaard, Sabrina Gallant, Michael Jeffrey, Shilpa Dogra

**Affiliations:** ^1^ Faculty of Health Sciences (Kinesiology) Ontario Tech University Oshawa Ontario Canada; ^2^ Faculty of Science and Engineering Technology Durham College Oshawa Ontario Canada

**Keywords:** angiogenesis, biomarkers, cardiovascular health, physical activity, sedentary behavior, walking

## Abstract

We aimed to determine salivary vascular endothelial growth factor (VEGF), epidermal growth factor (EGF), and angiogenin responses to prolonged and interrupted sitting in young, healthy adults. Each participant completed 4 h of prolonged sitting and two interrupted‐sitting sessions incorporating 3‐min walking breaks every 27 min at 35% or 50% of heart rate reserve, in random order. Saliva was collected immediately before and after each session and analyzed using enzyme‐linked immunosorbent assays. VEGF decreased from pre to post across all conditions (Pre: 0.63 ± 0.63; Post: 0.53 ± 0.67; *p* = 0.0013; partial *η* ^2^ = 0.09), while angiogenin increased (Pre: 7.42 ± 7.43; Post: 16.90 ± 12.80; *p* = 2.61 × 10^−12^; partial *η* ^2^ = 0.35), and EGF did not change (Pre: 6.14 ± 5.14; Post: 5.10 ± 4.37; *p* = 0.6885; partial *η* ^2^ = 0.0015). Time‐by‐condition interactions were not significant for any biomarker, indicating that light‐ and moderate‐intensity walking interruptions did not alter pre to post responses. These findings indicate that 4 h of sitting acutely lowers VEGF when interrupted by light‐ or moderate‐intensity walking, and elevates angiogenin in saliva; brief low‐to‐moderate walking breaks may be insufficient to modify responses in angiogenin. Future research should investigate whether higher‐intensity interruptions are needed to mitigate the effects of prolonged sitting.

## 1 INTRODUCTION

Cardiovascular disease is the leading cause of death worldwide and contributes substantially to reduced quality of life (Wu et al., 2023). Atherosclerosis, which involves the buildup of plaque on the inner walls of the arteries reducing blood flow, develops in part through endothelial dysfunction (Olvera Lopez et al., 2025; Wu et al., 2023). Normal endothelial function is supported by a range of vascular growth factors that promote angiogenesis, maintain vascular tone, and regulate immune function (Bruserud et al., 2005). Three factors relevant to this process are vascular endothelial growth factor (VEGF), epidermal growth factor (EGF), and angiogenin. VEGF is a potent angiogenic signal protein involved in vascular permeability and new vessel formation; EGF contributes to epithelial and vascular repair; and angiogenin has both angiogenic and anti‐inflammatory functions (Adair & Montani, 2010; Hastie et al., 2017; Karamysheva, 2008; Olsson et al., 2006; Stone et al., 2023). Dysregulation of these factors may contribute to impaired vascular adaptation, heightened inflammation, and endothelial dysfunction.

Exercise has been shown to promote the production of VEGF (Anastassios et al., 2021; Bizjak et al., 2021; Brandauer et al., 2013; Gavin et al., 2007; Kraus et al., 2004; Liu et al., 2025; Park et al., 2010; Saleh et al., 2020; Thorell et al., 2009; Wahl et al., 2011, 2013, 2014; Zuo et al., 2025), EGF (Fatouros et al., 2010), and Angiogenin (Bruserud et al., 2005). These increases occur even after a single bout of exercise, suggesting that frequent movement may benefit the maintenance of growth factor levels (Anastassios et al., 2021; Bizjak et al., 2021; Bruserud et al., 2005; Kraus et al., 2004; Kujach et al., 2020; Wahl et al., 2011, 2014; Zuo et al., 2025). On the other hand, excessive sedentary behavior, that is, any waking activity characterized by an energy expenditure ≤1.5 metabolic equivalents while in a sitting or reclining posture, is linked to a decrease in endothelial function (Tremblay et al., 2017) (Vandercappellen et al., 2022).

Experimental models of extreme sedentary behavior, such as bed rest or limb immobilization, have demonstrated reductions in VEGF bioavailability (Hyldahl et al., 2021; Navasiolava et al., 2010; Ringholm et al., 2011). For example, a significant decrease in plasma VEGF levels (51 ± 14% of baseline arbitrary units) was observed after 3 days of bed rest (Navasiolava et al., 2010). However, such models do not reflect typical sedentary patterns; more ecologically relevant paradigms, including prolonged sitting with or without interruptions, remain understudied. Recent exploratory work indicates that salivary VEGF, EGF, and angiogenin may respond to acute sitting bouts (Meens Miller et al., 2024), although methodological limitations, such as using pooled rather than individual samples, limit interpretation. Sex‐specific differences have also been reported, with some evidence suggesting females may experience attenuated vascular impairment during acute sitting, potentially due to the protective effects of estrogen (Credeur et al., 2019; Gotshall et al., 1994; Robert, 2023; Stanhewicz et al., 2018; Vranish et al., 2017; Waghorn et al., 2004). Understanding the physiological impact of prolonged and interrupted sitting is critical as population‐based data indicates that these patterns of sedentary behavior can change the risk of cardiovascular disease and related mortality (Onagbiye et al., 2024).

Research clearly demonstrates that growth factors increase following exercise and decrease in extreme models of sedentarism. However, it is unclear if similar patterns would be observed in ecologically relevant protocols of prolonged and interrupted sitting. Thus, the purpose of this study was to examine salivary VEGF, EGF, and angiogenin responses to 4 h of prolonged sitting, with and without walking interruptions, in young, healthy adults. Based on immobilization and bed rest studies (Hyldahl et al., 2021; Navasiolava et al., 2010; Ringholm et al., 2011), and preliminary salivary data (Meens Miller et al., 2024), we hypothesized that these growth factor levels would decline following prolonged sitting and that interruptions would mitigate such responses. We also explored sex differences in this work to determine whether future research needs to be powered for such analyses.

We chose to use saliva as it is a non‐invasive and easy to obtain biofluid suitable for repeated sampling during prolonged sitting protocols (Szabo et al., 2020). Furthermore, past research suggests that biomarkers such as VEGF, EGF, and angiogenin in the saliva originate from local production within salivary glands and oral immune cells, including acinar cells, ductal epithelial cells, and activated mast cells, highlighting saliva as a site of active biological signaling relevant to oral and systemic processes (Ding et al., 2024; Kulka et al., 2009; Pammer et al., 1998; Taichman et al., 1998; Thesleff et al., 1988). In this context, salivary concentrations of VEGF, EGF, and angiogenin should be interpreted as indicators rather than quantitative measures of tissue level growth factor production; these indicators provide insight into physiological responses to acute stressors of prolonged and interrupted sitting.

## 2 MATERIALS AND METHODS

### 2.1 Participants

Healthy young males and females aged 18–34 with a body mass index ≤30 kg/m^2^ were eligible to participate in the study. Those with current chronic disease (metabolic, respiratory, or cardiovascular), acute infection, musculoskeletal injury, pregnancy, recent dental surgery, or use of medications known to influence inflammatory or exercise responses were excluded. Participants provided written informed consent prior to participation. The study protocol was approved by the Ontario Tech University Research Ethics Board (REB# 17927). All procedures conformed to the standards set by the Declaration of Helsinki.

### 2.2 Study design and experimental protocol

A randomized crossover experimental design was used. Following a baseline session, participants completed three experimental conditions in randomized order. Session order was assigned using a computerized random number generator. Sessions were separated by 1 week and included: (1) prolonged sitting (PS): 4 h of prolonged sitting, (2) low‐intensity interrupted sitting (LI): 4 h of prolonged sitting interrupted by 3 min of walking at 35% heart rate reserve [HRR] every 27 min, and (3) moderate‐intensity interrupted sitting (MI): 4 h of prolonged sitting interrupted by 3 min of walking at 50% HRR every 27 min. During these sessions, participants were instructed to minimize lower‐limb movement while sitting and were permitted to engage in quiet seated activities (e.g., reading, computer use).

**FIGURE 1:**
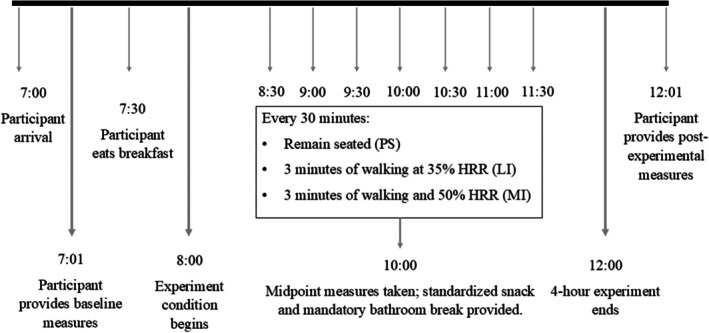
Session protocols (4‐h sitting interventions). Randomized crossover with three sessions: Prolonged Sitting (PS), Light‐Intensity interruptions (LI), and Moderate‐Intensity interruptions (MI). Each session lasted 240 min. In PS, participants sat continuously for 4 h; in LI and MI, participants sat continuously for 4 h, interrupted by 3‐min walks every 27 min at 35% HRR (LI) or 50% HRR (MI), respectively. Saliva was collected Pre (0 min) and Post (240 min). HRR, heart‐rate reserve; LI, light intensity; MI, moderate intensity; PS, prolonged sitting.

Participants were asked to avoid strenuous exercise, alcohol, smoking, and anti‐inflammatory medications 24 h prior to each session. To standardize energy intake during the experimental protocol, participants arrived fasted, without having consumed caffeine or supplements the morning of the session and consumed a light breakfast (protein shake and almonds) upon completing baseline measures. They were also provided a snack at 2 h (crackers and low‐sugar fruit juice box). All food items were prepackaged and identical across sessions to minimize dietary variability (450 kcal; 22.5 g fat, 40 g carbohydrate, 27 g protein). Participants arrived fasted (overnight) and were encouraged to drink ~1 L of water ~1 h before arrival. All sessions began between 07:00 and 09:00 in the same air‐conditioned laboratory, with room temperature maintained under typical indoor conditions. A mandatory bathroom break was included to ensure consistency between participants; participants were wheeled to the bathroom. All data collection occurred between May 2024 and May 2025. A detailed breakdown of the experimental sessions is shown in Figure 1.

### 2.3 Measures

The baseline session included measurement of resting heart rate and blood pressure (A&D Medical UA‐767FAM), height and body mass (Detecto Weigh Beam Eye‐Level), and maximal oxygen consumption (VO_2_max) using an incremental treadmill exercise test (Trackmaster, FullVision, Newton, KS) with continuous heart rate and gas exchange analysis (Parvo Medics 2400). Test termination criteria included volitional fatigue, VO_2_ plateau, respiratory exchange ratio ≥1.15, or HR within ±10 bpm of age‐predicted maximum. VO_2_max was defined as the highest oxygen uptake attained and verified by averaging five consecutive breaths around the peak value. Target heart rates for LI (35% HRR) and MI (50% HRR) walking bouts were calculated using the HRR method: (HRmax − HRrest) × intensity + HRrest.

### 2.4 Saliva sample collection and analysis

Saliva was collected pre (0 min) and post (240 min) experiment for each session using synthetic oral swabs (Salimetrics SalivaBio). Swabs were placed under the tongue or in the buccal cavity for 5 min, immediately centrifuged (VWR Clinical) at 1200 rpm for 5 min, and aliquoted into 300 μL vials stored at −80°C (Haier Biomedical). Prior to saliva collection, participants rinsed their mouths with water.

On the day of analysis, samples were thawed and centrifuged at 1500×*g* for 15 min at 4°C to remove mucins and particulates. Total protein concentration was determined via Bradford assay (Coomassie PLUS, Thermo Fisher Scientific) to normalize growth factor concentrations within saliva across participants. Samples were then analyzed using sandwich Enzyme‐Linked Immunoabsorbent Assays (ELISA) to examine EGF (Human EGF DuoSet ELISA, R&D Systems Inc.), VEGF (Human VEGF DuoSet ELISA, R&D Systems Inc.), and angiogenin (Human Angiogenin DuoSet ELISA, R&D Systems Inc.) following the manufacturer's protocol with absorbance measured at 450 nm using a Cytation 5 microplate reader (Bio‐Tek Instrumentation, VT, USA). All samples were assayed in duplicate, and normalized mean values were used. Concentrations below the assay limit of detection (LOD) were imputed using the formula LOD ÷ √2 × dilution factor (Croghan & Egeghy, 2003) which may slightly overestimate true values. This adjustment was applied to eight VEGF (LI post *n* = 3; MI pre *n* = 1; MI post *n* = 4) and seven angiogenin (PS pre *n* = 1; LI pre *n* = 3; MI pre *n* = 2; MI post *n* = 1) samples; no EGF samples fell below the LOD.

### 2.5 Statistical analysis

Baseline characteristics were summarized as means ± standard deviations for continuous variables and as counts and percentages for categorical variables. Model assumptions (normality of residuals and homoscedasticity) were assessed by visual inspection of residual plots, and biomarkers were analyzed on the log scale to stabilize variance.

Linear mixed‐effects models were fit by restricted maximum likelihood in R (RStudio version 2025.05.0 + 496) to examine pre‐post responses across conditions (PS, LI, MI). Biomarkers were analyzed on the log scale, with model assumptions checked by visual inspection of residuals. Values below the LOD followed assay‐log substitutions; remaining missing values were assumed missing at random and models used available cases. For VEGF, 5 possible observations were missing (PS pre *n* = 3; PS post *n* = 1; LI pre *n* = 1); for EGF, 9 possible observations were missing (PS pre *n* = 4; PS post *n* = 1; LI pre *n* = 2; MI pre *n* = 2); and for angiogenin, 6 possible observations were missing (PS pre *n* = 2; PS post *n* = 2; LI post *n* = 1; MI pre *n* = 1).

Fixed effects were timepoint (Pre, Post), condition (PS, LI, MI), sex (female, male), and the timepoint × condition interaction; a random intercept for participant accounted for the crossover design. Type III F‐tests used the Kenward‐Roger approximation for denominator degrees of freedom (Kenward & Roger, 1997). Estimated marginal means (EMMs) with 95% confidence intervals were obtained from the fitted mixed models and used for inference; figures showing box‐and‐whisker plots depict raw distributions (not model‐based CIs). Pre‐post differences within each condition were tested using Tukey‐adjusted pairwise contrasts. Statistical significance was accepted at *p* < 0.05. Effect sizes are reported as partial eta squared (partial *η* ^2^), with values of 0.01, 0.06, and 0.14 interpreted as small, moderate, and large, respectively.

## 3 RESULTS

Participant Characteristics. Twenty‐five participants were recruited; one withdrew due to scheduling conflicts, yielding a final sample of 12 females and 12 males. Participant characteristics are presented in Table 1.

**TABLE 1:** Participant characteristics of the final sample (*n* = 24; 12 females, 12 males). Values are presented as mean ± SD.

	Female (*n* = 12)	Male (*n* = 12)	Total sample (*n* = 24)
Age (years)	20.6 ± 3.7	23 ± 5.5	21.7 ± 4.7
Body mass index (kg/m^2^)	23.7 ± 3.0	23.8 ± 3.7	23.8 ± 3.3
Maximal aerobic capacity‐VO_2max_ (mL/kg^−1^/min^−1^)	36.5 ± 4.5	48.8 ± 6.3	42.6 ± 8.3

### 3.1 VEGF

**FIGURE 2:**
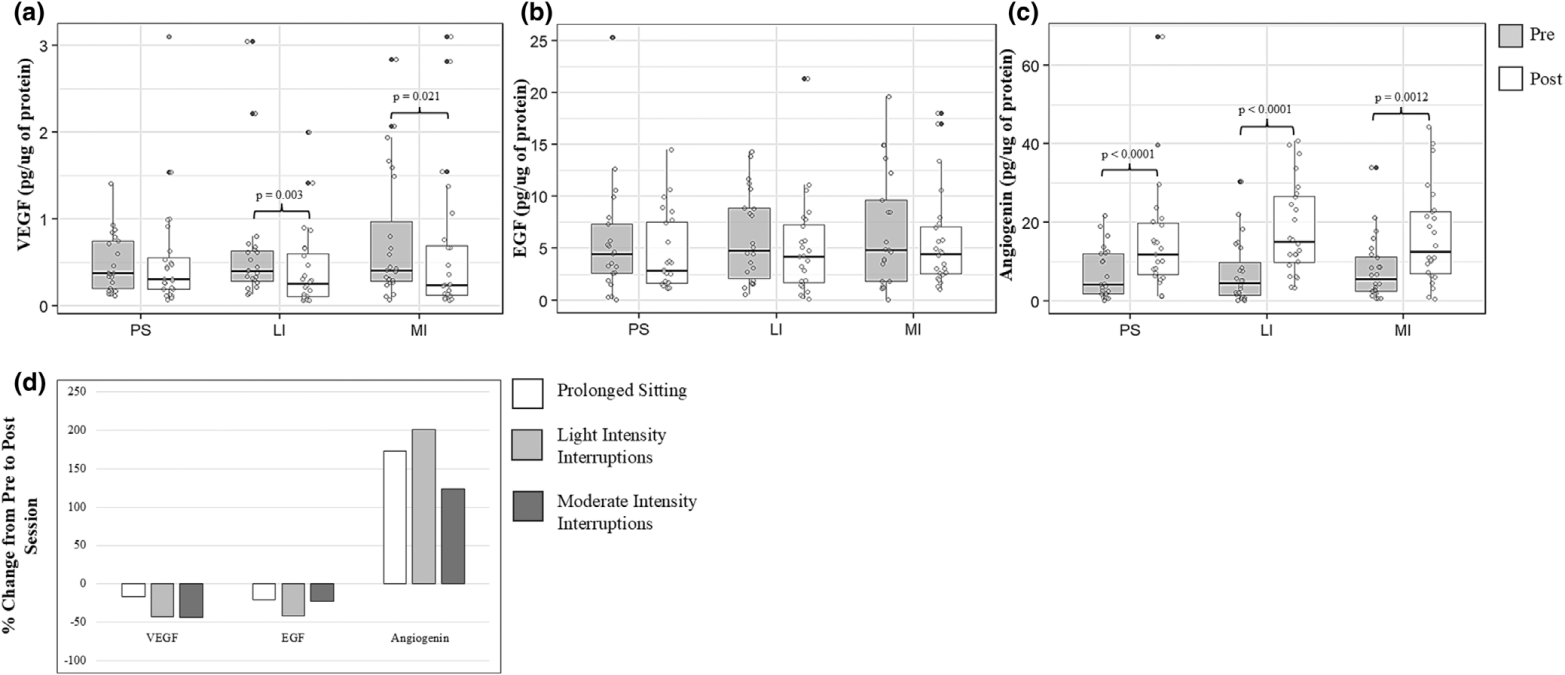
Changes in salivary growth factor concentrations during prolonged sitting (PS), low‐intensity (LI), and moderate‐intensity (MI) interruption sessions. Box‐and‐whisker plots display pre‐ (Pre, 0 h) and post‐session (Post, 4 h) concentrations for (a) VEGF, (b) EGF, and (c) angiogenin. Panel (d) shows pre‐post percent change for all three biomarkers. The line within each box represents the median; whiskers indicate minimum and maximum values. VEGF decreased significantly in LI and MI but not PS; EGF showed no significant changes; and angiogenin increased significantly in all conditions. No significant differences were observed between conditions in the magnitude of pre‐post change.

When averaging across conditions, a significant pre‐post decrease in VEGF concentrations was observed (Pre: 0.63 ± 0.63 pg μg^−1^; Post: 0.53 ± 0.67 pg μg^−1^; *F*(1, 111.5) = 10.95, *p* = 0.0013; partial *η* ^2^ = 0.09; Figure 2). When analyzed by condition, pre‐post decreases were significant in LI (Tukey‐adjusted *p* = 0.0026) and in MI (*p* = 0.021), with no significant change in PS (*p* = 0.708). The timepoint × condition interaction was not significant (*F*(2,111.2) = 1.86, *p* = 0.160). VEGF differed by sex (*F*(1,22.0) = 8.96, *p* = 0.0067; partial *η* ^2^ = 0.29), with higher concentrations observed in males than females when averaged across timepoints and conditions.

### 3.2 EGF

When averaging across conditions, no significant pre‐post change in EGF concentrations was observed (Pre: 6.14 ± 5.14 pg μg^−1^; Post: 5.10 ± 4.37 pg μg^−1^; *F*(1,109.4) = 0.16, *p* = 0.6885; partial *η* ^2^ ≈ 0.001). When analyzed by condition, no significant pre‐post differences were observed in PS (*p* = 0.713), LI (*p* = 0.235), or MI (*p* = 0.907) after Tukey adjustment. The timepoint × condition interaction was not significant (*F*(2,109.2) = 0.70, *p* = 0.498). EGF differed by sex (*F*(1,21.7) = 24.50, *p* = 6.73 × 10^−5^; partial *η* ^2^ = 0.52), with higher concentrations observed in males than females when averaged across timepoints and conditions.

### 3.3 Angiogenin

When averaging across conditions, a significant pre‐post increase in angiogenin concentrations was observed (Pre: 7.42 ± 7.43 pg μg^−1^; Post: 16.90 ± 12.80 pg μg^−1^; *F*(1,113.15) = 61.59, *p* = 2.61 × 10^−12^; partial *η* ^2^ = 0.35). When analyzed by condition, pre‐post increases were significant in PS (Tukey‐adjusted *p* < 0.0001), LI (*p* < 0.0001), and MI (*p* = 0.0012). The timepoint × condition interaction was not significant (*F*(2,113.1) = 1.71, *p* = 0.186). Angiogenin differed by sex (*F*(1,22.0) = 5.65, *p* = 0.0265; partial *η* ^2^ = 0.21), with higher concentrations observed in males than females when averaged across timepoints and conditions.

Sex‐differences for VEGF, EGF, and angiogenin are shown in Figures S1–S3.

## 4 DISCUSSION

We sought to determine the acute responses of VEGF, EGF, and angiogenin to prolonged and interrupted sitting protocols in young, healthy adults. Our results indicate that 4 h of sitting led to a decrease in VEGF and an increase in angiogenin, whereas EGF remained unchanged. For VEGF, decreases were observed during light and moderate walking interruption sessions only, while for angiogenin, changes observed during prolonged sitting were not altered by light‐ or moderate‐intensity walking interruptions. To our knowledge, this is the first study to examine VEGF, EGF, and angiogenin responses to prolonged sitting using a randomized crossover design, contributing new insight into the salivary biomarkers associated with sitting‐induced vascular changes.

We hypothesized that all three growth factors would decrease following prolonged sitting, which aligns with previous work in our laboratory using average pixel density (Meens Miller et al., 2024). We also hypothesized that walking interruptions would attenuate a decrease in growth factors. However, no significant differences were observed between conditions, suggesting that the walking interruptions used here may not have been sufficient to modify endothelial growth factor responses. Given that both interruption protocols involved low‐moderate exercise intensities (35% and 50% HRR, respectively), the lack of change may reflect insufficient intensity to induce angiogenic signaling. This interpretation is supported by previous work. For example, Liu et al. (2025) reported that short bursts of high‐intensity aerobic exercise (80% VO_2_max) elicit larger VEGF responses than moderate‐intensity exercise (60% VO_2_max) (Liu et al., 2025). Similarly, Dogra et al. (2019) and O'Rourke et al. (2023) showed that high‐intensity cycling sprints prevent pro‐inflammatory IL‐8 increases, whereas moderate walking only attenuates them. These findings, along with our results, suggest that a higher intensity of interruption may be needed to attenuate the negative physiological responses to acute bouts of prolonged sitting.

Although VEGF decreased from pre‐ to post‐session when averaged across conditions, no significant change was observed during prolonged sitting; decreases occurred only in the interrupted sitting conditions, and no time‐by‐condition interactions were observed. One possible explanation for this unexpected observation is that muscle interstitial VEGF responses to movement do not translate directly to parallel changes in salivary VEGF, reflecting preferential utilization and redistribution to muscle during walking bouts and temporal dynamics between active tissue and saliva (Hoier & Hellsten, 2014; Tang et al., 2010). It is also possible that a 4‐h sitting period was too short to provoke a measurable decrease in salivary VEGF alone, while brief interruptions were sufficient to induce a response in active skeletal muscle. Future research should examine more frequent sampling points over longer sessions to capture potential temporal shifts in VEGF dynamics. More broadly, salivary growth factor concentrations should not be interpreted as directly reflecting tissue‐level production, as they encompass downstream signaling between blood and saliva.

The increase in angiogenin was unexpected, as we hypothesized that it would decrease in response to prolonged sitting. This could be explained by the role of angiogenin in immune and inflammatory responses (Sheng & Xu, 2016). Angiogenin is thought to have anti‐inflammatory properties, as its serum protein concentrations tend to increase in response to stress (Sheng & Xu, 2016). Previous research shows that prolonged sitting promotes several pro‐inflammatory biomarkers (Benatti & Ried‐Larsen, 2015; Dogra et al., 2019; Hamilton et al., 2008; O'Rourke et al., 2023). Thus, it is possible that angiogenin increased in response to prolonged sitting due to its anti‐inflammatory function rather than a proangiogenic factor.

The lack of significant differences in EGF concentrations across conditions was also not as hypothesized. We had anticipated that EGF concentrations would decrease following prolonged sitting, and walking interruptions would mitigate this response. The lack of significant differences may be related to the diverse physiological roles of EGF. Beyond its role in vascular regulation, EGF is broadly involved in cellular proliferation and repair across multiple tissues, including in saliva (Wong & Guillaud, 2004). Thus, local tissue repair and turnover processes may obscure small vascular‐related changes in salivary EGF levels. Since the role of salivary EGF and the mechanisms regulating its concentration are not well understood, it is also possible that changes occurred but were not captured within our sampling window.

A main effect of sex was observed for all three growth factors, with higher concentrations in males when averaged across timepoints and conditions; however, these findings are exploratory, as the study was not powered for sex‐based inferences. Future research is needed to further explore these sex differences, as it may have implications for exercise‐based recommendations for prevention and management of pre‐clinical risk factors for cardiovascular disease. Although, such recommendations are premature. Salivary vascular growth factors may be useful as comparative indicators for evaluating the impact of experimental movement protocols, but have not been sufficiently developed or researched for use as clinical markers.

### 4.1 Strengths

First, only a few studies have examined growth factors in sedentarism. Prior work has examined VEGF responses to sedentarism in the context of bedrest or complete limb immobilization (Hyldahl et al., 2021; Navasiolava et al., 2010; Ringholm et al., 2011) while Meens Miller et al. (2024) examined VEGF, EGF and angiogenin during sitting but without individual‐level differences (Meens Miller et al., 2024). This study helps to fill these gaps by examining the responses of growth factors to acute bouts of prolonged sitting at the individual level. Second, salivary biomarkers are known to exhibit high inter‐individual variability due to factors such as flow rate, circadian influences, and genetic or hormonal differences (Chiappin et al., 2007; Surdu et al., 2025; Yoshizawa et al., 2013). The use of a randomized crossover design and linear mixed‐effects modeling helped account for this variability while preserving participant‐specific patterns, strengthening confidence that observed changes reflect true biological responses to sitting and interruptions.

### 4.2 Limitations

This sample consisted of young, healthy adults, limiting generalizability to the overall population due to the effects of various factors such as age, disease status, and body mass (Accattato et al., 2017; Bruserud et al., 2005; Luttrell et al., 2021; MacNeil et al., 2021). We were unable to find robust salivary data on VEGF, EGF, or angiogenin for sample size calculations; instead, we used salivary IL‐8 concentrations to guide recruitment targets, which resulted in relatively small effect sizes and low power. This also meant we were underpowered for examination of sex differences. High inter‐individual variability in salivary responses may have further amplified variance within this small sample, limiting sensitivity to subtle effects (Chiappin et al., 2007). Another limitation of this work is the lack of objective measurement of fidgeting during the PS sessions. While a researcher was present during each session to monitor the participant, it is possible that some participants fidgeted more during the session than others. Future research can use a leg strain gauge to monitor such minor movements for comparison (Dogra et al., 2019). Future research in this area may also need to focus on individual differences. Our preliminary work suggests this approach could highlight important determinants of the response to prolonged sitting (Figures S4–S6).

### 4.3 Conclusion

In conclusion, across the three sessions, we observed a decrease in VEGF and an increase in angiogenin, while EGF remained unchanged. For angiogenin, brief light‐ and moderate‐intensity walking breaks did not significantly alter the increase observed during 4 h of sitting, indicating that such interruptions may be insufficient to counteract the vascular effects of prolonged sitting. For VEGF, a decrease was observed during interrupted walking but not prolonged sitting. Future research should examine interruptions of varying intensity and duration to determine whether a higher dose of interruption is necessary to disrupt physiological changes associated with prolonged sitting. Studies exploring whether repeated daily exposures result in changes in salivary biomarker levels over time are also needed.

## AUTHOR CONTRIBUTIONS

Alicia Kollaard was responsible for data curation, formal analysis, investigation, methodology, visualization, and writing of the original draft. Sabrina Gallant was responsible for formal analysis, investigation, methodology, and writing (reviewing and editing). Michael Jeffrey was responsible for data curation, methodology, and writing (reviewing and editing). Shilpa Dogra was responsible for conceptualization, funding acquisition, project administration, resources, supervision, and writing (reviewing and editing). All authors interpreted the data, revised the manuscript for important intellectual content, and approved the final version.

## FUNDING INFORMATION

This work was supported by the Natural Sciences and Engineering Research Council of Canada (Discovery Grant # RGPIN‐2023‐03543 to Dr. Shilpa Dogra) and Ontario Tech University.

## CONFLICT OF INTEREST STATEMENT

The authors declare no conflicts of interest.

## Supporting information


**Figure S1.** Changes in VEGF concentrations during prolonged sitting, low‐intensity, and moderate‐intensity interruption sessions by sex.
**Figure S2.** Changes in EGF concentrations during prolonged sitting, low‐intensity, and moderate‐intensity interruption sessions by sex.
**Figure S3.** Changes in Angiogenin concentrations during prolonged sitting, low‐intensity, and moderate‐intensity interruption sessions by sex.

**Figure S4.** Individual changes in salivary VEGF across conditions, by sex.
**Figure S5.** Individual changes in salivary EGF across conditions, by sex.
**Figure S6.** Individual changes in salivary Angiogenin across conditions, by sex.

## Data Availability

Data generated or analyzed during this study are available from the corresponding author upon reasonable request.
