# Supplementary material for: Salivary vascular growth factor responses to prolonged and interrupted sitting in young, healthy adults
Source: Physiol Rep. 2026 Feb 23;14(4):e70798. doi: 10.14814/phy2.70798 (PMC12929194; doi:10.14814/phy2.70798)
Supplement: Supplementary file 1 — Figure S1. Changes in VEGF concentrations during prolonged sitting, low‐intensity, and moderate‐intensity interruption sessions by sex. Figure S2. Changes in EGF concentrations during prolonged sitting, low‐intensity, and moderate‐intensity interruption sessions by sex. Figure S3. Changes in Angiogenin concentrations during prolonged sitting, low‐intensity, and moderate‐intensity interruption sessions by sex. [file PHY2-14-e70798-s002.pdf]

Figure S1. Changes in VEGF concentrations during prolonged sitting, low-intensity, and moderate-intensity interruption sessions by sex..

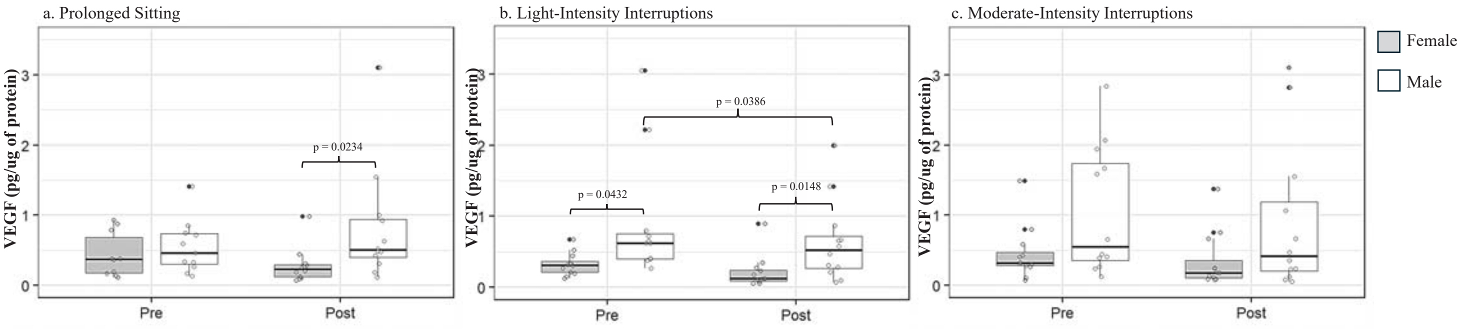

Legend: Box-and-whisker plots display pre- (Pre, 0h) and post-session (Post, 4 h) concentrations for VEGF by sex. The line within each box represents the median; whiskers indicate minimum and maximum values.

Figure S2. Changes in EGF concentrations during prolonged sitting, low-intensity, and moderate-intensity interruption sessions by sex..

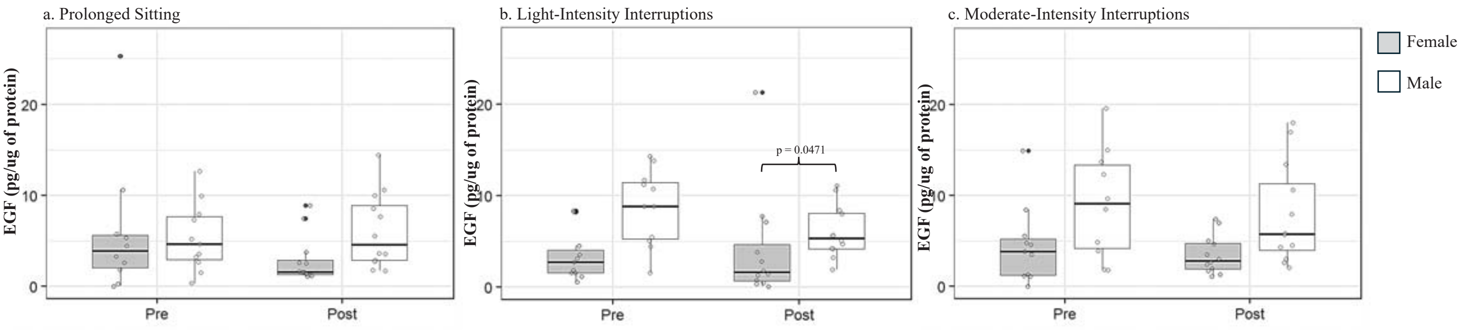

Legend: Box-and-whisker plots display pre- (Pre, 0h) and post-session (Post, 4 h) concentrations for EGF by sex. The line within each box represents the median; whiskers indicate minimum and maximum values.

Figure S3. Changes in angiogenin concentrations during prolonged sitting, low-intensity, and moderate-intensity interruption sessions by sex..

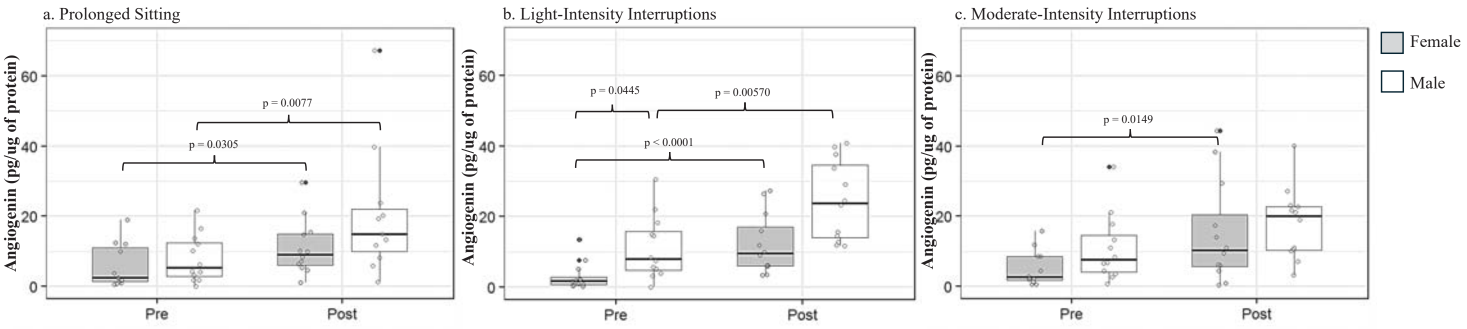

Legend: Box-and-whisker plots display pre- (Pre, 0h) and post-session (Post, 4 h) concentrations for angiogenin by sex. The line within each box represents the median; whiskers indicate minimum and maximum values.
