# Supplementary material for: Salivary vascular growth factor responses to prolonged and interrupted sitting in young, healthy adults
Source: Physiol Rep. 2026 Feb 23;14(4):e70798. doi: 10.14814/phy2.70798 (PMC12929194; doi:10.14814/phy2.70798)
Supplement: Supplementary file 2 — Figure S4. Individual changes in salivary VEGF across conditions, by sex. Figure S5. Individual changes in salivary EGF across conditions, by sex. Figure S6. Individual changes in salivary Angiogenin across conditions, by sex. [file PHY2-14-e70798-s001.pdf]

Figure S4: Individual changes in salivary VEGF across conditions, by sex.

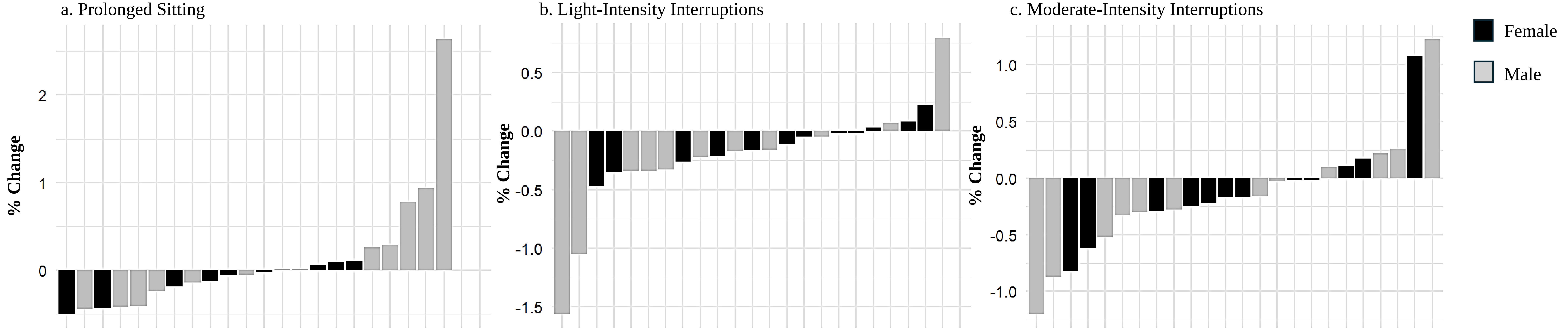

Legend: Waterfall plots display change in concentrations of VEGF from pre- (0h) and post-session (4h) by sex.

Figure S5: Individual changes in salivary EGF across conditions, by sex.

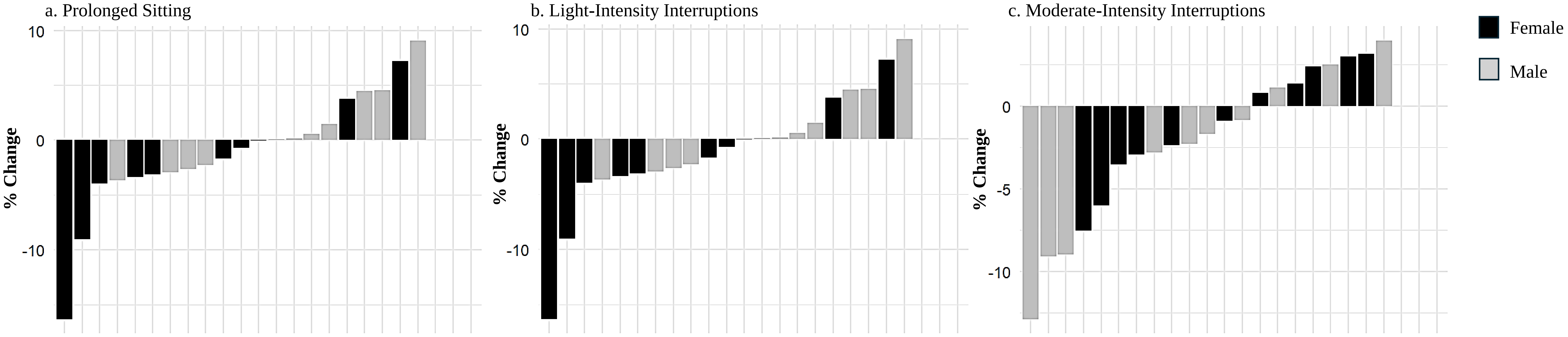

Legend: Waterfall plots display change in concentrations of EGF from pre- (0h) and post-session (4h) by sex.

Figure S6: Individual changes in salivary angiogenin across conditions, by sex.

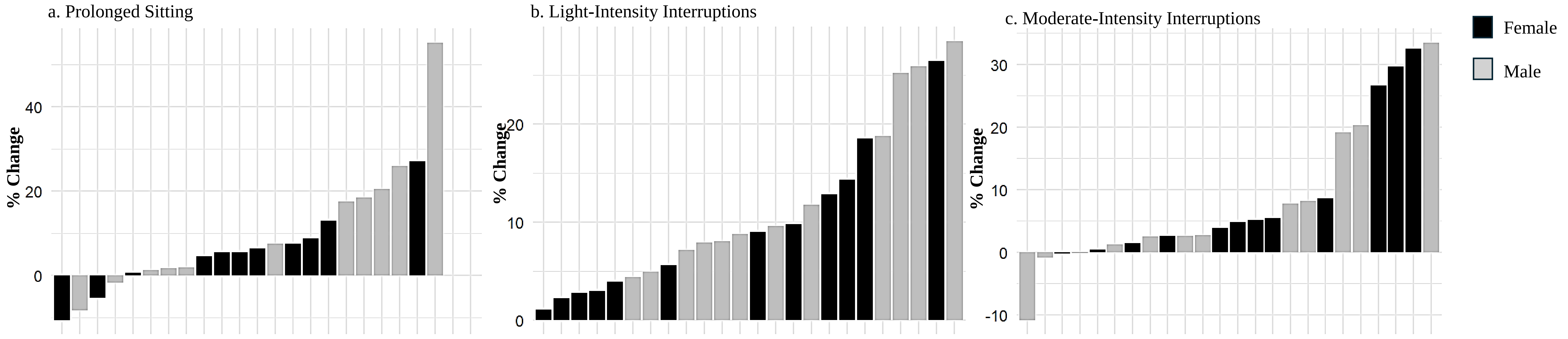

Legend: Waterfall plots display change in concentrations of angiogenin from pre- (0h) and post-session (4h) by sex.
